# Productive Germinal Center Responses Depend on the Nature of Stimuli Received by Anti-Insulin B Cells in Type 1 Diabetes–Prone Mice

**DOI:** 10.4049/immunohorizons.2300036

**Published:** 2023-06-01

**Authors:** Dudley H. McNitt, Bryan A. Joosse, James W. Thomas, Rachel H. Bonami

**Affiliations:** * Division of Rheumatology and Immunology, Department of Medicine, Vanderbilt University Medical Center, Nashville, TN; † Department of Pathology, Microbiology and Immunology, Vanderbilt University Medical Center, Nashville, TN

## Abstract

Islet autoantibodies, including those directed at insulin, predict type 1 diabetes (T1D) in mice and humans and signal immune tolerance breach by B lymphocytes. High-affinity insulin autoantibodies and T follicular helper cell involvement implicate germinal centers (GCs) in T1D. The V_H_125^SD^ BCR transgenic model, in which 1–2% of peripheral B lymphocytes recognize insulin, enables direct study of insulin-binding B cells. Our prior studies showed that anti-insulin B cell receptor transgene site-directed to H chain locus mice fail to generate insulin Ab following T-dependent immunization, but it was unclear whether anti-insulin B cells were blocked for GC initiation, survival, or differentiation into Ab-secreting cells. Here, we show that insulin-binding B cells in T1D-prone anti-insulin B cell receptor transgene site-directed to H chain locus mice can spontaneously adopt a GC phenotype and undergo class switching to the IgG1 isotype, with little if any switching to IgG2b. T-dependent immunizations with insulin SRBC or insulin CFA drove anti-insulin B lymphocytes to adopt a GC phenotype, despite blunted insulin Ab production. Dual immunization against self (insulin) and foreign (4-hydroxy-3-nitrophenylacetyl hapten conjugated to keyhole limpet hemocyanin) Ags showed an anti-insulin (but not anti-4-hydroxy-3-nitrophenylacetyl) Ab block that tracked with increased expression of the apoptosis marker, activated caspase 3, in self-reactive GC B cells. Finally, T-independent immunization with insulin conjugated to *Brucella abortus* ring test Ag released immune tolerance to allow robust expansion of anti-insulin GC B cells and IgG-switched insulin Ab production. Overall, these data pinpoint GC survival and Ab-secreting cell differentiation as immune tolerance blocks that limit T-dependent, but not T-independent, stimulation of anti-insulin B cell responses.

## Introduction

Type 1 diabetes (T1D) results from the autoimmune destruction of β cells within the islets of Langerhans in the pancreas. CD8^+^ T cells mediate β cell destruction, a process that is supported by other immune cells, including B cells (1–3). An essential role for B cells in T1D is demonstrated by findings that therapeutic B cell depletion prevents T1D development in NOD mice and preserves β cell function in individuals with new-onset T1D (4, 5). Moreover, the presence of B cell infiltrate in the pancreas is associated with more aggressive T1D development in humans (6). B cells contribute to T1D pathogenesis via Ag presentation of islet autoantigens, such as insulin, to cognate CD4^+^ T cells (7–9).

Insulin is a major autoantigen targeted by the immune system in NOD mice and humans (10). B cell recognition of insulin is necessary for NOD mice to develop diabetes; NOD mice that harbor an increased frequency of anti-insulin B cells due to the V_H_125 BCR transgene develop accelerated T1D, whereas transgenic mice lacking this critical B cell specificity are protected (11, 12). Therapy that specifically depletes anti-insulin B cells reduces diabetes development in NOD mice (13). Diabetes development was observed in transgenic NOD mice with an IgM-restricted anti-insulin H chain transgene (V_H_125Tg), but not in mice that expressed VH281Tg, which differed only by lacking two key amino acid mutations necessary for insulin recognition, highlighting the importance of anti-insulin B cells in T1D (11). These data also show that accelerated T1D development can occur even when limited anti-insulin IgG is present, due to IgM isotype restriction by this non-site-directed/class-switch-incompetent V_H_125Tg (7, 14, 15). The finding that anti-insulin B cells can present autoantigen to T cells in vitro and support an Ab-independent role in driving T1D development (7) is consistent with studies showing that B cell–specific deletion of the diabetogenic MHC class II molecule, IA^g7^, is also disease protective in NOD mice (8).

Insulin autoantibodies that predict T1D are often high affinity, a characteristic associated with germinal center (GC) derivation (16). In line with this, GC anti-insulin B cells, but not non-GC anti-insulin B cells from anti-insulin B cell receptor transgene site-directed to H chain locus (V_H_125^SD^.NOD) mice, process and present the unique insulin peptide register recognized by one of the major anti-insulin T cell specificities identified in mice and humans that develop T1D (17–20). This anti-insulin B/T collaboration results in immune tolerance breach that allows anti-insulin B cell differentiation into GC B cells and insulin autoantibody-secreting cells (17). Tertiary lymphoid structures and ectopic GCs form spontaneously within the islets of NOD mice (21). Anti-CD40 stimulation (which mimics T cell help) drives anti-insulin B cells in V_H_125^SD^. NOD mice to proliferate normally; yet, T cell-dependent (TD) immunization fails to drive anti-insulin IgG production (15). This block in insulin Ab production suggests that anti-insulin B cells fail to undergo class switch, fail to enter or survive in GCs, and/or fail to differentiate into Ab-secreting cells.

To address these possibilities, we assessed the ability of anti-insulin B cells to form GCs spontaneously and following TD and T cell-independent (TI) immunization. We found that anti-insulin B cells form spontaneous GCs in multiple lymphoid tissues and within the pancreas, as well as undergo class switch. TD immunization fails to induce insulin Ab production; yet, it can drive anti-insulin B cells to adopt a GC phenotype, depending on the TD adjuvant used. In the setting where anti-insulin GC development is blunted, expression of the apoptosis marker activated caspase 3 is increased. In contrast, TI immunization leads to the formation of productive GCs and insulin Ab production, with no changes in apoptosis marker expression with respect to non-insulin-binding GCs. These findings highlight (1) anti-insulin B cell differentiation into Ab-secreting cells and (2) anti-insulin B cell GC survival after select TD immunization as key immune tolerance checkpoints that limit anti-insulin Ab production.

## Materials and Methods

### Animals

NOD.129P2(Cg)-Igh^tm1.1Jwt^/J (V_H_125^SD^.NOD) mice that contain an anti-insulin BCR H chain site directed to the H chain locus were generated as previously described (15). V_H_125^SD^.NOD mice were backcrossed to NOD for at least 20 generations; the mice used in all experiments were heterozygous for the anti-insulin V_H_125^SD^ BCR H chain. Conventional NOD (NOD/ShiLtJ) mice were obtained from The Jackson Laboratory (Bar Harbor, ME) and maintained in our colony. All mice were housed under specific pathogen-free conditions and given autoclaved food and water. All studies were approved by the Institutional Animal Care and Use Committee of Vanderbilt University Medical Center, fully accredited by the Association for Assessment and Accreditation of Laboratory Animal Care.

### Cell isolation

Cells were isolated from spleens, medial iliac lymph nodes, pancreatic lymph nodes, mesenteric lymph nodes, and pancreas as previously described (22, 23). Briefly, spleens were freshly harvested and macerated through a 70-µm cell strainer with HBSS + 10% bovine calf serum (BCS), cells were pelleted, RBCs were lysed with an RBC lysis solution (140 mM NH_4_Cl, 17 mM Tris), and cells were resuspended in FACS buffer (1× PBS + 1 mM EDTA + 10% FBS). Lymph nodes were processed similarly without RBC lysis. Pancreata were digested with 3 ml of 1 mg/ml collagenase P (Sigma-Aldrich) diluted in HBSS and incubated at 37°C with shaking for 30 min, then tissue was disrupted with an 18-gauge needle. HBSS + 10% BCS was immediately added to inhibit continued collagenase activity. Cells were washed in HBSS and resuspended in FACS buffer for downstream flow cytometry staining and analysis.

### Abs and flow cytometry

Cells were stained for flow cytometric analysis using the following murine reactive Abs and reagents: CD19-BUV373 (clone 1D3), CD19-PE (clone 1D3), B220-BUV395 (clone RA3-6B2), B220-PacBlue (clone RA3-6B2), GL7-FITC (clone GL7), IgD-Alexa Fluor 350 (clone 11-26c), IgM-Alexa Fluor 680 (clone AF6-78), streptavidin-allophycocyanin, streptavidin-BV421 (BD Biosciences); rabbit anti-mouse cleaved caspase 3, goat anti-rat-IgG F(ab′)-PE and goat anti-rabbit-IgG F(ab’)-Alexa Fluor 647 from Cell Signaling Technology; CD16/CD32 (FC block, clone 2.4G2), and viability dye e510 (Tonbo Biosciences). Biotinylated human insulin (Sigma-Aldrich) was generated and used to detect insulin-binding specificity, whereas insulin-occupied BCRs were detected via biotinylated anti-insulin mAb123 (HB-123; American Type Culture Collection) as described previously (11, 24). 4-Hydroxy-3-nitrophenyl acetyl (NP)-allophycocyanin was used to detect NP-binding specificity (25). To prepare fluorescently labeled NP, PhycoPro allophycocyanin (Agilent, Santa Clara, CA) was dialyzed against 3% sodium bicarbonate overnight at 4°C. A 40 µg/ml solution of NP-Osu (4-hydroxy-3-nitrophenylacetic acid succinimide ester; Biosearch Technologies, Novato, CA) in dimethylformamide was added to the allophycocyanin solution and rocked at room temperature for 2 h. The solution was first dialyzed against 3% sodium bicarbonate solution and then against 1× PBS and stored at 4°C. Flow cytometry data were acquired using a BD Biosciences LSR II or a BD Biosciences LSRFortessa flow cytometer, and data were analyzed using FlowJo software (BD Biosciences).

### Preparation of insulin SRBC conjugate

SRBCs were conjugated to insulin as described previously (26), with the following modifications. Insulin was washed with 0.1 M bicine saline and incubated with M-maleimidobenzoyl-*N*-hydroxy-succinimide (Sigma-Aldrich) in dimethylformamide for 1 h at room temperature with gentle mixing. Acylated insulin was precipitated on ice with citrate phosphate buffer, pH 5, washed, and resuspended in a solution of bicine saline. Packed defibrinated SRBCs (Remel, San Diego, CA) were washed with bicine saline and incubated in bicine saline containing 2-iminothiolane for 30 min at room temperature. Thiolated SRBCs were washed three times in bicine saline and then conjugated to modified insulin for 1 h at room temperature and washed with sterile 1× PBS. Recombinant human insulin (Sigma-Aldrich) was conjugated to *Brucella abortus* ring test Ag (BRT; U.S. Department of Agriculture Animal and Plant Health Inspection Services, Ames, IA) as previously described (27, 28).

### Immunizations

Preimmune sera were collected from 8–13-wk-old male and female prediabetic V_H_125^SD^.NOD mice. For TD immunizations, mice were immunized s.c. bilaterally at the base of the tail, with 25 μg insulin B9-23 peptide (NovoPro Bioscience) mixed with 25 μg of 4-hydroxy-3-nitrophenylacetyl hapten conjugated to keyhole limpet hemocyanin (NP-KLH; Biosearch Technologies, Novato, CA) emulsified in CFA. Sera were harvested at 3 wk following immunization, after which mice were boosted with 10 μg B10-23 insulin/25 μg NP-KLH emulsified in IFA s.c. bilaterally at the base of the tail. Tissues and sera were collected 7 d after boost. For TD immunization, insulin SRBCs (prepared as above) or SRBCs were packed via centrifugation, washed once with 1× PBS, and resuspended in 1 ml of 1× PBS. Mice were each immunized s.c. bilaterally at the base of the tail with 100 µl of a 1:10 dilution of washed SRBCs. Tissues and sera were collected 10 d after immunization. For TI immunizations with BRT and insulin BRT, mice were immunized s.c. bilaterally at the base of the tail; tissues and sera were collected 5 d after immunization.

### ELISA

Competitive binding in ELISA was used to detect insulin-specific Abs, as outlined previously (15). Briefly, 96-well MaxiSorp Nunc plates (Thermo Scientific) were coated with 1 μg/ml human insulin in borate-buffered saline overnight at 37°C. Sera were diluted 1:100 in 1× PBS, and plates were incubated either for 1 h at room temperature or overnight at 4°C. To calculate insulin-specific OD, parallel samples were incubated in the presence of 100 µg/ml human insulin, and values were subtracted from noninhibited wells to calculate inhibitable OD and indicate insulin-specific binding. Total IgG was detected with goat anti-mouse IgG conjugated to alkaline phosphatase (catalog number 1030-04; SouthernBiotech). Plates were washed, then incubated with substrate solution (10 μg/ml *p*-nitrophenyl phosphate substrate [Sigma-Aldrich] in potassium carbonate and magnesium chloride buffer). OD was read at 405 nm using a microplate autoreader (Bio-Tek). For IgG1 and IgG2a allotype detection, goat anti-mouse IgG1^[a]^ (clone, 10.9, BD Biosciences), IgG2a^[a]^ (clone 8.3, BD Biosciences), IgG1^[b]^ (clone B68-2, BD Biosciences), or IgG2a^[b]^ conjugated to biotin (clone 5.7, BD Biosciences) was incubated for 1 h at room temperature. Plates were then washed and incubated with avidin conjugated to alkaline phosphatase (catalog number E2636; Sigma-Aldrich) for 1 h at room temperature and then developed with substrate as outlined above. All washes used 1× PBS containing 0.5% Tween 20.

NP-specific IgG was measured as previously described (29). Briefly, 96-well MaxiSorp Nunc plates were coated with 1 µg/ml NP_32_-BSA (Biosearch Technologies, Middlesex, UK) in 1× PBS overnight at 4°C. Wells were blocked with PBS containing 0.5% BSA and 0.5% Tween 20. Sera were diluted 1:100 in 1× PBS, and plates were incubated for 1 h at room temperature and developed with substrate as outlined above.

### Statistics

Statistical tests used for each experiment are indicated in the corresponding figure legends, and significance values were calculated using Prism (GraphPad Software).

## Results

### Anti-insulin B cells in V_H_125^SD^.NOD can develop spontaneous germinal centers and undergo isotype switch

**FIGURE 1.**
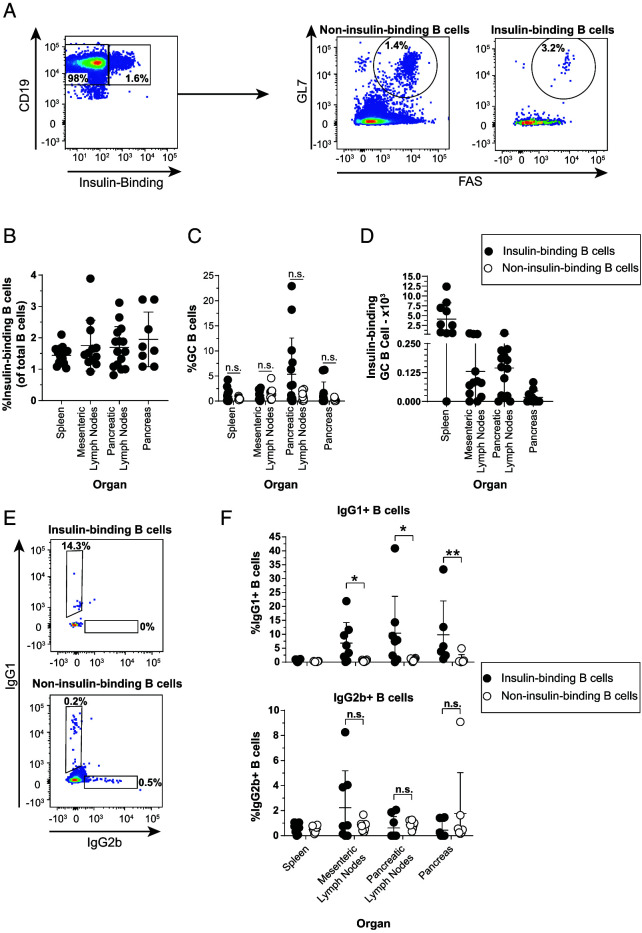
Anti-insulin B cells in V_H_125^SD^.NOD can develop spontaneous GCs and undergo IgG class switch. Spleen, mesenteric lymph nodes, pancreatic lymph nodes, and pancreas were harvested from female V_H_125^SD^.NOD mice, and cells were stained for flow cytometric analysis of GC and IgG subset markers. (**A**) Representative flow cytometry plots identifying GC B cells (B220^+^, CD19^+^, live, GL7^high^, FAS^high^) among insulin-binding and non-insulin-binding B cells in pancreatic lymph nodes. The frequency of (**B**) insulin-binding B cells among total B cells across each organ. The frequency of Ag-binding B cells (**C**) or the number of insulin-binding GC B cells (**D**) is shown. (**E**) Representative flow cytometry plots identifying class-switched cells among insulin-binding and non-insulin-binding B cells (B220^+^, CD19^+^, live) from pancreatic lymph nodes. (**F**) The frequency of IgG1^+^ (top) and IgG2b^+^ (bottom) among insulin-binding and non-insulin-binding B cells identified as in (D) is shown. The insulin-binding GC B cell frequency (C), cell number (D), and class-switched insulin-binding B cell frequency (F) are shown for mice that had >20 insulin-binding B cells in the parent gate. Eight- to seventeen-wk-old female NOD mice were used. *n* ≥ 6 mice per group, *n* ≥ 3 experiments (A–C) and *n* ≥ 3 mice per group, *n* ≥ 2 experiments (D and E). *p < 0.05, **p < 0.01, Mann-Whitney *U* test.

The failure of V_H_125^SD^.NOD mice to produce insulin autoantibodies could be explained by an inability of insulin-binding B cells to enter spontaneous GCs. We therefore used flow cytometry to determine if insulin-binding B cells can adopt a GC phenotype in V_H_125^SD^. NOD mice, as outlined in Fig. 1A. Although insulin-binding B cells were readily detected in spleen, lymph nodes, and pancreas (Fig. 1B), anti-insulin GC (GL7^hi^ FAS^hi^) B cells were identified more sporadically across mice, suggesting that anti-insulin GC formation is a rare or transient phenomenon (Fig. 1C, 1D). A similar frequency of insulin-binding and non-insulin-binding B cells adopted a GC phenotype in spleen and mesenteric lymph nodes, with some individual mice demonstrating a higher frequency of insulin-binding GC B cells in the pancreatic draining lymph nodes (Fig. 1C).

The two major isotypes of spontaneous insulin autoantibody detected in wild-type (WT) NOD mice are IgG1 and IgG2b (30); however, V_H_125^SD^.NOD mice produce very little anti-insulin IgG spontaneously (15). To test whether anti-insulin B cells are class switching to IgG1 or IgG2b but failing to differentiate into Ab-secreting cells in V_H_125^SD^.NOD mice, we used flow cytometry (Fig. 1E). A greater proportion of anti-insulin B cells were IgG1^+^ in the mesenteric lymph nodes, pancreatic lymph nodes, and pancreas relative to non-insulin-binding B cells (Fig. 1F, top). Conversely, IgG2b^+^ B cells were less frequently observed, and no statistical difference between the anti-insulin and non-insulin-binding B cell populations was present in any tissue examined (Fig. 1F, bottom). Thus, our data demonstrate that despite a block in spontaneous insulin autoantibody production, insulin-binding cells displayed a GC phenotype and expressed the class-switched IgG1 isotype in several tissues.

### T-dependent immunization with insulin SRBCs elicits poor anti-insulin Ab production but drives anti-insulin germinal center B cell expansion

Transgenic anti-insulin B cells in V_H_125^SD^.NOD mice fail to produce anti-insulin Abs in response to immunization with the immunodominant insulin B-chain peptide (B9:23) mixed with CFA; what little anti-insulin IgG was detected was produced by nontransgenic/endogenous (b allotype) B cells and was of the IgG2a^b^ isotype, whereas IgG1 (a or b allotype) was not observed (15). SRBCs are potent drivers of TD immunity, and direct conjugation of insulin to SRBCs is known to drive anti-insulin Ab production in WT NOD mice (26). To assess whether this alternative TD adjuvant could drive productive anti-insulin GCs in V_H_125^SD^.NOD mice, we immunized mice s.c. with insulin conjugated to SRBCs (insulin SRBCs) or SRBCs alone as a control. Medial iliac lymph nodes and sera were collected 10 d after immunization.

**FIGURE 2.**
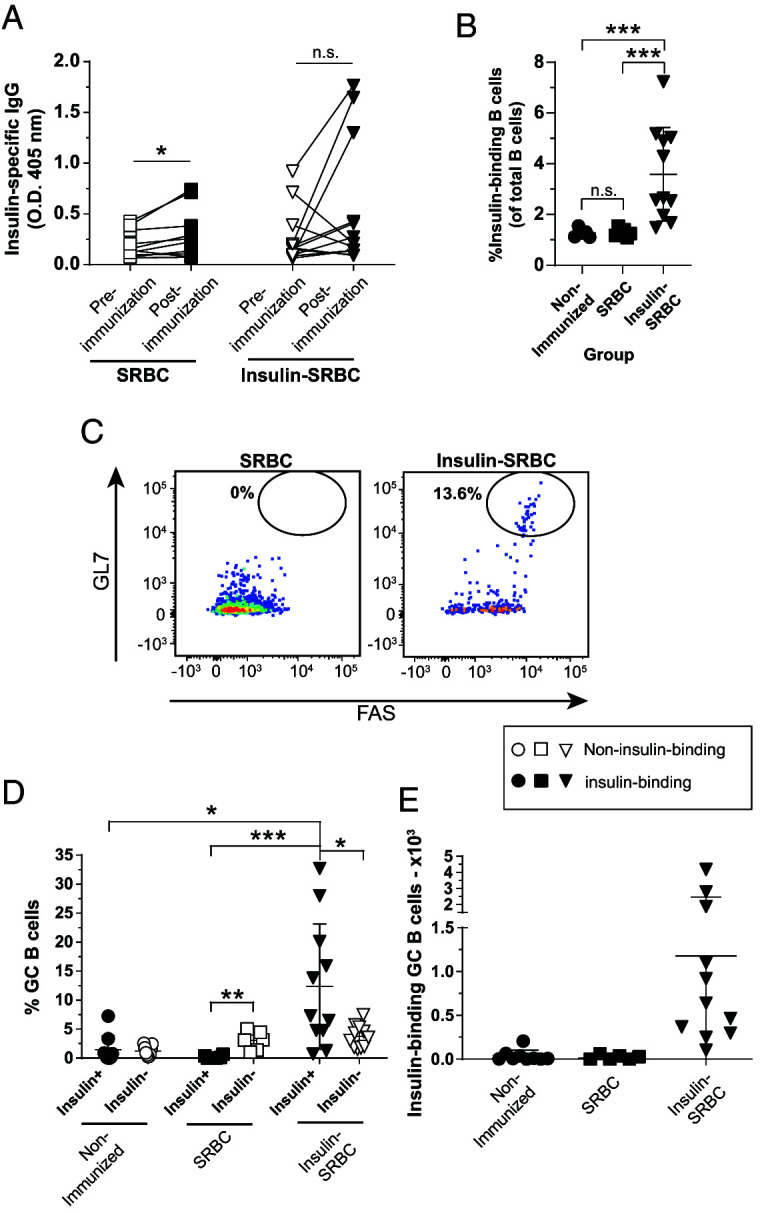
TD immunization with insulin SRBCs elicits poor anti-insulin Ab production but drives anti-insulin GC B cell expansion. V_H_125^SD^.NOD mice were immunized s.c. at the base of the tail with either SRBCs or insulin conjugated to SRBCs (insulin SRBCs) as described in *Materials and Methods*. Draining medial iliac lymph nodes and sera were harvested 10 d after immunization. (**A**) Serum anti-insulin Ab production was measured by ELISA in V_H_125^SD^.NOD mice before and after immunization with either insulin SRBC or SRBC. The frequency of (**B**) insulin-binding B cells among total B cells after immunization with either SRBC or insulin SRBCs. (**C**) Representative flow cytometry plots identify GC B cells as in Fig. 1 among insulin-binding B cells in draining lymph nodes of mice immunized with SRBCs (left) and insulin SRBCs (right). The (**D**) frequency (**E**) and number of GC B cells among insulin-binding B cells between nonimmunized, SRBC-, and insulin SRBC–immunized mice are shown for mice that had >20 insulin-binding B cells in the parent gate. Eight- to fourteen-wk-old male and female NOD mice were used. *n* ≥ 6 mice per group, *n* ≥ 2 experiments. *p < 0.05, **p < 0.01, ***p < 0.001, paired two-tailed *t* test (A) and Mann-Whitney *U* test (B).

Insulin SRBC immunization resulted in inconsistent insulin Ab production; although some mice elicited a clear insulin Ab response, the majority of mice failed to elicit a clear response (Fig. 2A). We attribute this heterogeneous response to biological variability between mice, because an agglutination assay used to confirm insulin was robustly conjugated to SRBCs (data not shown). Furthermore, for each of the two experiments performed for this immunization study, we confirmed immunogenicity of insulin SRBCs via anti-insulin IgG production in at least one mouse per group. It is possible the anti-insulin IgG detected in a few mice arose from endogenous B cells, which might also help explain the variation in an immune response to an autoantigen known to evoke functional silencing via anergy (15). SRBCs alone lead to a small increase in anti-insulin IgG production, whereas insulin SRBC immunization resulted in inconsistent insulin Ab production (Fig. 2A).

Insulin SRBC immunization resulted in a significant increase in the total frequency of anti-insulin B cells in the medial iliac lymph nodes compared with nonimmunized and SRBC-immunized controls (Fig. 2B). Additionally, the frequency and number of anti-insulin B cells that adopted a GC phenotype was significantly increased after insulin SRBC immunization compared with nonimmunized and SRBC controls and compared with non-insulin-binding B cells in insulin SRBC–immunized mice (Fig. 2C–2E). Immunization with control SRBCs alone did not yield a significant increase in anti-insulin GC B cell formation compared with non-insulin-binding B cells, as expected (Fig. 2D, 2E). These results demonstrate that TD insulin SRBC immunization drives anti-insulin B cell acquisition of a GC phenotype, despite failing to fully reverse their functional silencing to drive insulin Ab production.

### T-dependent immunization with CFA/insulin peptide elicits limited Ab production and germinal center formation compared with foreign Ag responses in the same V_H_125^SD^.NOD mice

**FIGURE 3.**
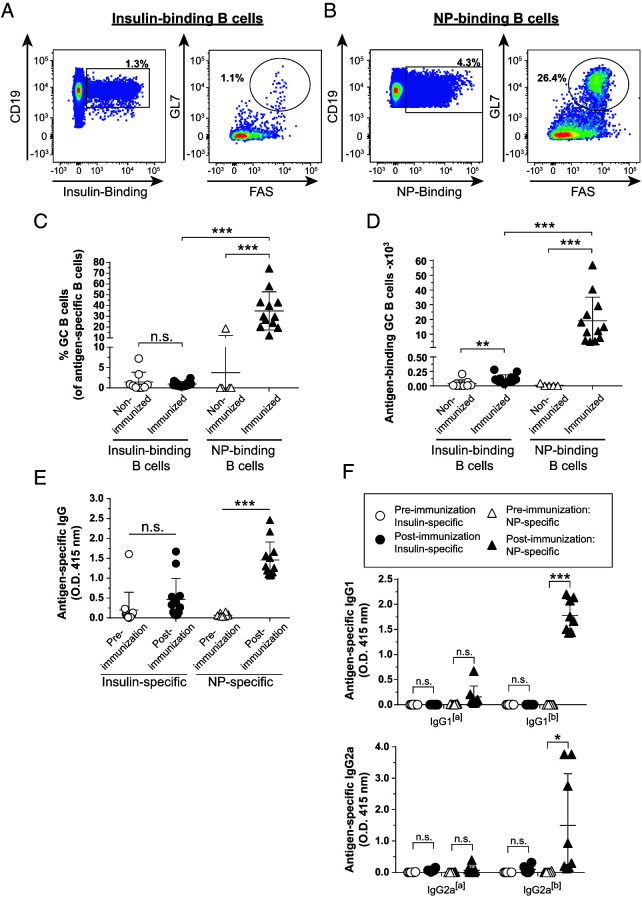
TD immunization with CFA/insulin peptide elicits limited Ab production and GC formation compared with foreign Ag responses in the same V_H_125^SD^.NOD mice. V_H_125^SD^.NOD mice were immunized s.c. with both insulin B chain peptide B:9-23 and NP-KLH emulsified in CFA as described in *Materials and Methods*. Three weeks later, mice were boosted with insulin B chain peptide B:9-23 and NP-KLH emulsified in IFA. Draining medial iliac lymph nodes and sera were collected before immunization or 7 d after boost. (**A** and **B**) Representative flow cytometry plots identify GC B cells as in Fig. 1 among insulin-binding (A) and NP-binding B cells (B) in lymph nodes harvested from immunized mice. Frequency (**C**) or number (**D**) of Ag-binding GC B cells is shown. The GC B cell frequency is shown for mice that had >20 insulin-binding or NP-binding B cells in the parent B cell gate. Anti-insulin and anti-NP Ab production from both preimmunization and postboost measured in sera by ELISA in V_H_125^SD^.NOD mice. (**E**) Total Ag-specific IgG or (**F**) allotype-specific Ag-specific IgG1 (top) or IgG2a (bottom) indicates transgenic (IgG1^[a]^ or 2a^[a]^) or endogenous (IgG1^[b]^ or IgG2a^[b]^) B cell origin. Ten- to fifteen-wk-old male and female NOD mice. *n* ≥ 6 mice per group, *n* ≥ 3 experiments. *p < 0.05, **p ≤ 0.01, *** *p* ≤ 0.001, two-tailed *t* test (E and F) or Mann-Whitney *U* test (C and D).

Anti-insulin B cells unexpectedly adopted a GC phenotype following TD immunization with SRBCs, despite limited insulin Ab production. To test whether this also occurs with a different TD adjuvant, we immunized mice s.c. with a mixture of insulin B:9-23 peptide emulsified in CFA. The foreign Ag, NP-KLH, was included in this immunization to rule out the possibility that V_H_125^SD^.NOD mice show an overall blunted TD response, even to foreign Ag. Three weeks later, mice were boosted with B:9-23 and NP-KLH emulsified in IFA. Medial iliac lymph nodes and sera were collected 7 d after boost. Insulin-binding and NP-binding B cells were readily detected in immunized mice (Fig. 3A, 3B). Immunization increased the frequency of NP-binding, but not insulin-binding, B cells that adopted a GC phenotype (Fig. 3C; *p* < 0.001 and *p* = 0.37, respectively). A dramatic increase in the number of NP-binding GC B cells was observed, with only a modest increase in the number of insulin-binding GC B cells compared with nonimmunized mice (Fig. 3D; *p* < 0.001 and *p* = 0.002, respectively). TD immunization with B:9-23 + NP-KLH in CFA did not result in a significant increase in the frequency of insulin-binding B cells, whereas an increase in the frequency of NP-binding B cells was observed compared with nonimmunized mice (data not shown).

V_H_125^SD^.NOD mice produced NP-specific Abs in response to CFA TD immunization (Fig. 3E; *p* < 0.0001), but an anti-insulin Ab response was not detected (Fig. 3E; *p* = 0.18). ELISA using allotype-specific reagents against two different IgG isotypes elicited by NP-KLH/CFA immunization in other models (31) showed the majority of NP-specific IgG detected was derived from the endogenous, nontransgenic cells [b] as compared with the V_H_125^SD^ transgene [a] (Fig. 3F). Overall, these data demonstrate that the limited endogenous repertoire in V_H_125^SD^.NOD mice can support the formation of productive NP-specific GCs and IgG with CFA immunization, whereas insulin-binding B cells show limited differentiation into GC B cells or insulin Ab-secreting cells in response to this TD adjuvant.

### T-independent immunization results in anti-insulin Ab production and robust anti-insulin germinal center B cell expansion

Our previous studies support the concept that TI stimuli and immunization can promote anti-insulin B cell responses, whereas stimuli mimicking T cell help and TD immunization often elicit blunted responses (14, 15, 28). BRT shares many characteristics with other classic type 1 TI Ags (27, 32). Insulin conjugated to BRT (insulin BRT) drives anti-insulin B cells to form robust GCs and produce anti-insulin IgG of the IgG2c, but not IgG1, allotype in nonautoimmune V_H_125^SD^.C57BL/6 (B6) mice (28). To test whether TI immunization could similarly breach tolerance in V_H_125^SD^.NOD mice, we immunized mice s.c. with insulin BRT or BRT alone. Medial iliac lymph nodes and sera were collected 5 d after immunization.

**FIGURE 4.**
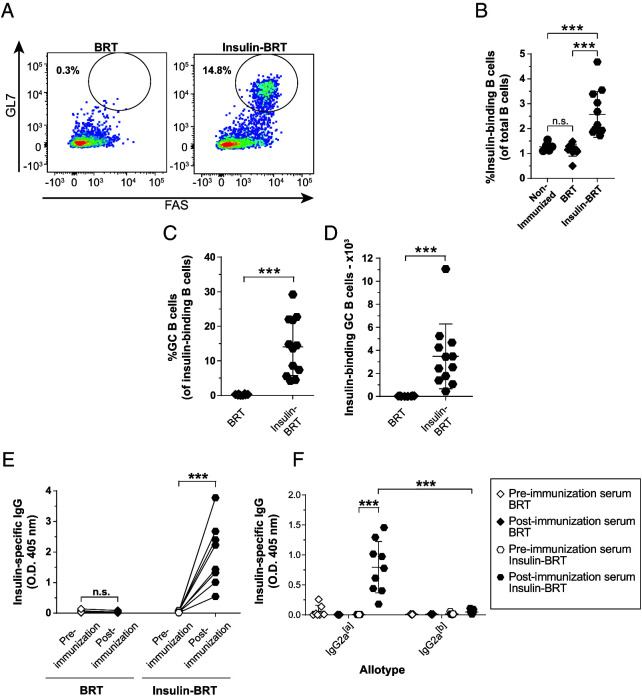
TI immunization results in anti-insulin Ab production and robust anti-insulin GC B cell expansion. V_H_125^SD^.NOD mice were immunized with the TI immunogen, BRT, or insulin-conjugated BRT (insulin BRT) s.c. at the base of the tail as described in *Materials and Methods*. Draining medial iliac lymph nodes and sera were harvested 5 d after immunization. (**A**) Representative flow cytometry plots identify GC B cells among insulin-binding B cells as in Fig. 1 in draining lymph nodes of mice immunized with BRT (left) and insulin BRT (right). The frequency of (**B**) insulin-binding B cells among total B cells after immunization with either BRT or insulin BRT. The frequency (**C**) and number (**D**) of GC B cells among insulin-binding B cells between nonimmunized, BRT and insulin BRT immunized mice are plotted for *n* ≥ 6 mice per group, *n* ≥ 2 experiments. The insulin-binding GC B cell frequency (C) is shown for mice that had >20 insulin-binding B cells in the parent B cell gate. (**E** and **F**) Anti-insulin Ab was measured by ELISA in V_H_125^SD^.NOD sera before and after immunization with BRT alone or insulin BRT (*n* ≥ 7 mice plotted per group). (E) Total insulin-specific IgG or (F) allotype-specific anti-insulin IgG2a indicates transgenic (IgG2a^[a]^) or endogenous (IgG2a^[b]^) B cell origin. All mice were 8–14-wk-old male and female NOD mice. ***p ≤ 0.001, two-tailed *t* test (D and E) or Mann-Whitney *U* test (B and C).

To assess the impact of TI immunization on anti-insulin GC formation, lymph nodes were isolated from unimmunized and BRT insulin–immunized mice and stained for GC markers (Fig. 4A). Insulin BRT resulted in a significant increase in the total frequency of anti-insulin B cells compared with nonimmunized and BRT-immunized negative controls (Fig. 4B). Insulin BRT immunization drove significantly higher frequencies (Fig. 4C; *p* < 0.0001) and numbers (Fig. 4D; *p* < 0.0001) of insulin-specific GC B cells compared with control BRT-immunized mice.

As opposed to BRT alone, immunization with insulin BRT elicited high levels of insulin Ab compared with preimmunization sera (Fig. 4E). Immunization with insulin BRT elicited high levels of transgene-derived anti-insulin IgG2a (the NOD equivalent to IgG2c in C57BL/6 mice) compared with preimmune V_H_125^SD^.NOD mice (Fig. 4F). These data demonstrate that transgene-derived anti-insulin B cells in V_H_125^SD^.NOD mice can break anergy to produce functional GCs and differentiate into Ab-secreting cells through TI immunization.

### Abortive anti-insulin GC B cells show increased activated caspase 3 expression relative to foreign Ag-specific GC B cells

**FIGURE 5.**
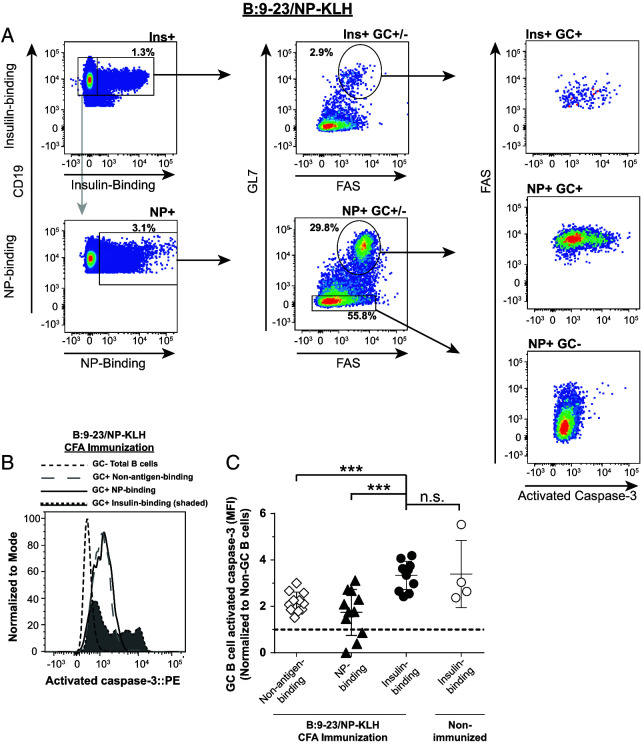
Abortive anti-insulin GC B cells show increased activated caspase 3 expression relative to foreign Ag-specific GC B cells. V_H_125^SD^.NOD mice were immunized s.c. with both insulin B chain peptide B:9-23 and NP-KLH emulsified in CFA as in Fig. 3. Draining medial iliac lymph nodes and sera were collected from unimmunized mice or 7 d postboost (B:9-23/NP-KLH CFA). (**A**) Representative flow cytometry plots show activated caspase 3 expression (right) among Ag-specific GC B cells among insulin-binding (top left) and NP-binding B cells (bottom left) in lymph nodes harvested from B:9-23+NP-KLH/CFA-immunized mice. GC B cells were identified in Fig. 1 with the exception that cells were not excluded on the basis of viability dye staining. Negative control non-GC NP-binding B cells are also shown. (**B**) Representative histogram overlay comparing activated caspase 3 expression in insulin-binding GC B cells to NP- and non-insulin-binding GC B cells after immunization. GC B cells are shown as a negative staining reference. Data were normalized to mode to account for differences in cell numbers of each group. (**C**) Normalized activated caspase 3 MFI between insulin-binding, NP-binding, or non-Ag-binding GC B cells after immunization. *n* ≥ 4 mice per group plotted from *n* ≥ 2 experiments. Non-GC total B cells activated caspase 3 MFI values were used for normalization within each mouse. Dotted line shows no change from this reference. Activated caspase 3 MFI of anti-insulin GC B cells isolated from the same lymph nodes of nonimmunized mice are shown as an additional control. Eight- to fourteen-wk-old male and female NOD mice were used. ***p ≤ 0.001, Mann-Whitney *U* test.

Peripheral tolerance mechanisms can prevent the expansion and differentiation of autoreactive B cells into memory and plasma cells (33). Apoptosis is a normal part of the GC reaction, which, when defective, can contribute to autoreactive B cell expansion and autoantibody production (34–36). This led us to postulate that the failure of anti-insulin GCs to expand after insulin peptide/CFA immunization in V_H_125^SD^. NOD mice (Figs. 2, 3) might be due to an increase in apoptosis of anti-insulin B cells within the GC. To test this, we immunized mice as outlined in Fig. 3 and measured activated caspase 3, a marker of apoptosis (34, 37), in Ag-specific GC B cells (Fig. 5A). To account for any variation in activated caspase 3 staining intensity across experiments, the mean fluorescence intensity (MFI) of GC B cells was normalized to non-GC B cells present in the same mice, which contain little activated caspase 3 (NP-binding non-GC B cells [Fig. 5A, 5B]; insulin binding non-GC data not shown) (34). Insulin-binding GC B cells had significantly higher expression of activated caspase 3 (normalized MFI average ratio, 3.3) compared with NP-binding GC B cells (normalized MFI average ratio, 1.7) and compared with non-NP or insulin Ag-binding GC B cells (“non-antigen-binding”; normalized MFI average ratio, 2.2) (Fig. 5C). Interestingly, there was no statistical difference in activated caspase 3 expression between insulin-binding GC B cells from immunized mice compared with anti-insulin GC B cells that formed spontaneously in nonimmunized mice (normalized MFI average ratio, 3.4) (Fig. 5C). These data demonstrate that during insulin peptide/CFA immunization, where anti-insulin GC B cells fail to undergo much expansion, activated caspase 3 expression is increased in autoreactive anti-insulin GC B cells compared with foreign Ag (NP)-binding GC B cells. This implies that reduced anti-insulin B cell survival in CFA-induced GCs may be a contributing factor to the inability of anti-insulin B cells to mount a productive insulin Ab response in this immunization setting.

### Anti-insulin germinal center B cells that undergo expansion following immunization do not show an increase in activated caspase 3 expression

**FIGURE 6.**
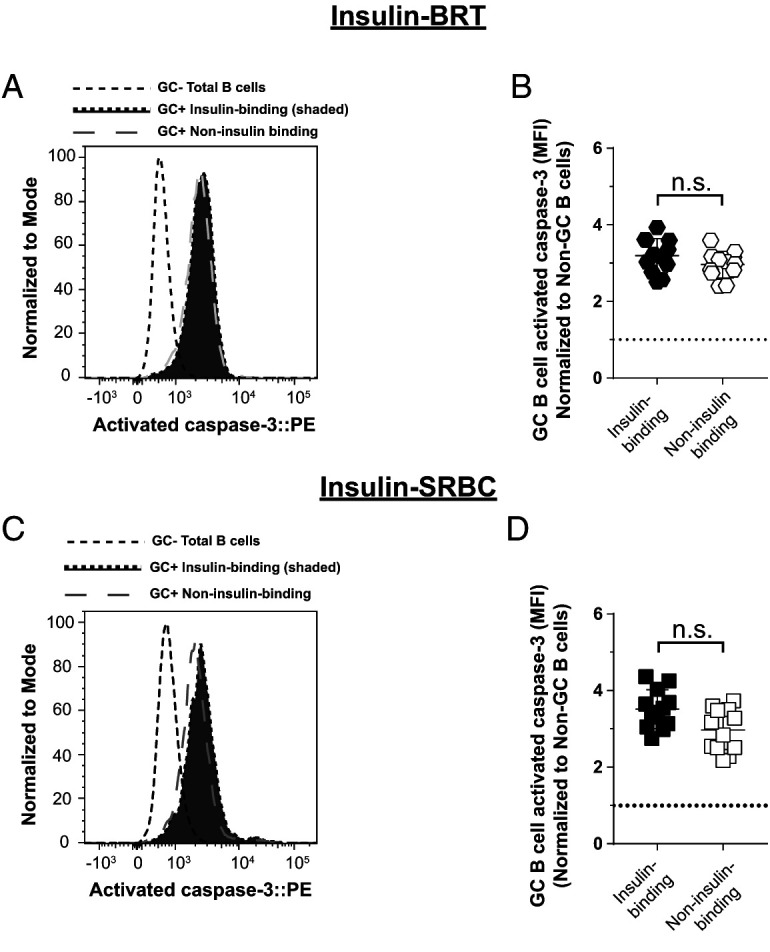
Anti-insulin GC B cells that undergo expansion following immunization do not show an increase in activated caspase 3 expression. V_H_125^SD^.NOD mice were immunized s.c. at the base of the tail with insulin conjugated to SRBCs (insulin SRBCs) as in Fig. 3. Draining medial iliac lymph nodes and sera were harvested 10 d after immunization. (**C** and **D**) V_H_125^SD^.NOD mice were immunized with insulin-conjugated BRT (insulin BRT) s.c. at the base of the tail as in Fig. 4. Draining medial iliac lymph nodes and sera were harvested 5 d after immunization. (**A** and C) Representative histogram overlays comparing activated caspase 3 expression in insulin-binding GC B cells to non-insulin-binding GC B cells after insulin SRBC immunization (A) or after insulin BRT immunization (C). GC B cells are shown as a negative staining reference. Data were normalized to mode to account for differences in cell numbers of each group. (**B** and D) Normalized activated caspase 3 MFI of insulin-binding and non-insulin-binding GC B cells after insulin SRBC immunization (B) or insulin BRT immunization (D). *n* ≥ 4 mice per group are plotted from *n* ≥ 2 experiments. Non-GC total B cell activated caspase 3 MFI values were used for caspase normalization within each mouse. Dotted line shows no change from this reference. Eight- to fourteen-wk-old male and female NOD mice were used. Mann-Whitney *U* test.

Anti-insulin GC expansion occurs following TI immunization against insulin BRT (Fig. 4) and TD immunization against insulin SRBCs (Fig. 2), but not in response to TD immunization with insulin CFA (Fig. 3). Given our observed increase in activated caspase 3 expression in anti-insulin GC B cells relative to foreign Ag-specific GC B cells following CFA immunization, we next assessed whether insulin-binding B cells showed increased activated caspase 3 expression under conditions where anti-insulin GC B cell expansion was observed (i.e., insulin BRT and insulin SRBC immunizations). Insulin-binding GC B cells in insulin SRBC–immunized mice were not significantly different in their expression of activated caspase 3 (normalized MFI average ratio, 3.5) compared with non-insulin-binding GC B cells present in the same mice (normalized MFI average ratio, 3.0) (Fig. 6A, 6B). Similarly, there was no statistical difference in insulin-binding GC B cell expression of activated caspase 3 in insulin BRT–immunized mice (normalized MFI average ratio, 3.2) compared with non-insulin-binding GC B cells in these mice (normalized MFI average ratio, 3.0) (Fig. 6C, 6D). Together with findings in Fig. 5, our data support the concept that anti-insulin B cell expansion and GC formation, as observed after insulin BRT and insulin SRBC immunization, does not lead to an increase in apoptotic anti-insulin B cells, whereas failure to expand and form GCs, such as after immunization with CFA, tracks with elevated expression of the apoptosis marker activated caspase 3.

### Spontaneous anti-insulin germinal center B cells have elevated activated caspase 3 expression compared with non-insulin-binding germinal center B cells

**FIGURE 7.**
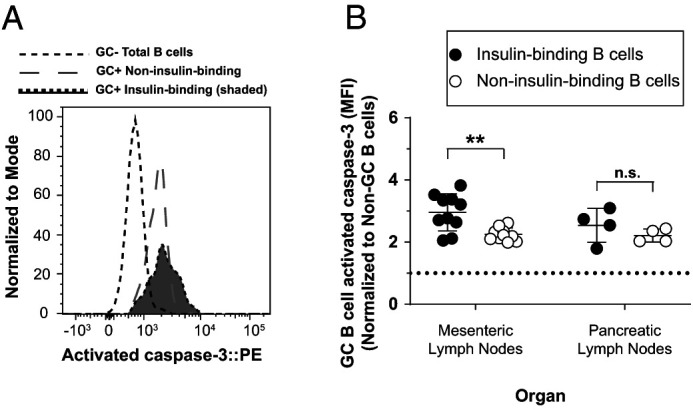
Spontaneous anti-insulin GC B cells have elevated activated caspase 3 expression compared with non-insulin-binding GC B cells. Mesenteric lymph nodes and pancreatic draining lymph nodes were harvested from female V_H_125^SD^.NOD mice, and activated caspase 3 staining was measured among GC B cells (identified as in Fig. 5). (**A**) Representative histogram overlay comparing activated caspase 3 expression in insulin-binding GC B cells and non-insulin-binding GC B cells in mesenteric lymph nodes. GC total B cells are shown as a negative staining reference. Data were normalized to mode to account for differences in cell numbers of each group. (**B**) Normalized activated caspase 3 MFI is shown for insulin-binding and non-insulin-binding GC B cells within each organ. Each dot represents one mouse. Activated caspase 3 MFI was normalized to non-GC total B cell activated caspase 3 MFI. Dotted line indicates no change from this reference. For all experiments, 8–14-wk-old female NOD mice were used. *n* ≥ 6 mice per group, *n* ≥ 2 experiments. **p ≤ 0.01, Mann-Whitney *U* test.

Spontaneous GCs can develop and are elevated in autoimmune prone mouse models compared with nonautoimmune prone mice (38, 39). To determine if activated caspase 3 expression differs between autoreactive and nonautoreactive GC B cells that form spontaneously, we examined caspase 3 expression in both the pancreatic and mesenteric lymph nodes. Mesenteric lymph nodes were included because they are rich with spontaneous GCs, even in nonautoimmune mice (40). Mesenteric lymph nodes provide an important control that allows us to investigate GC B cells that form independently of T1D autoimmunity, providing a fairer comparison across insulin-binding and non-insulin-binding GC B cells. Using a similar phenotyping strategy as in Figs. 5 and 6, we found that the average normalized activated caspase 3 MFI ratio was significantly higher in insulin-binding GC B cells than in non-insulin-binding GC B cells that were present in the mesenteric lymph nodes in unimmunized V_H_125^SD^.NOD mice (Fig. 7A, 7B). The average normalized activated caspase 3 MFI ratio was marginally increased in insulin-binding GC B cells compared with non-insulin-binding GC B cells in pancreatic draining lymph nodes, although this did not reach statistical significance (*p* = 0.3, two-tailed Student *t* test). Therefore, anti-insulin B cells are prone to undergo apoptosis to a greater degree than non-insulin-binding B cells.

## Discussion

Anti-insulin V_H_125^SD^.NOD B cells drive accelerated T1D, despite producing little if any anti-insulin Ab either spontaneously or following TD immunization with insulin-CFA (15). This suggests that an immune tolerance block is imposed on insulin-binding B cells, albeit one that fails to control diabetes. Here, we show that this block is not imposed at GC induction, because anti-insulin B cells can adopt a GC phenotype spontaneously and following both TI and TD immunization. These findings are consistent with our previous report that anti-insulin B cells isolated from V_H_125^SD^.NOD mice can proliferate normally to anti-CD40, a stimulus that mimics T cell help, as well as the TI stimulus LPS (15). Rather, we show that the block in insulin Ab production is at the point of insulin-binding B cell differentiation into Ab-secreting cells and, for the case of insulin CFA immunization, also in enhanced anti-insulin GC B cell apoptosis.

Insulin autoantibodies that form spontaneously in WT NOD mice are of the IgG1 and IgG2b isotypes (30). Insulin-binding B cells in V_H_125^SD^.NOD mice spontaneously underwent class switching to IgG1, and less so to IgG2b, albeit sporadically (Fig. 1F). Although IgG2b can fix complement, IgG1 does not and shows weaker binding to activating Fc receptors relative to IgG2b (41). Thus, immune tolerance mechanisms may be acting to limit anti-insulin B cell class switch to the more inflammatory IgG2b isotype. Given that NOD mice are deficient in the complement protein C5 (42) and express functionally important polymorphisms in the inhibitory FcγRIIb expressed on B cells (43), this complex topic will require careful investigation in the future. Anti-insulin B cells in V_H_125^SD^-8F10.NOD mice are released from this immune tolerance block to spontaneously produce IgG1, IgG2b, and IgG2a autoantibody when anti-insulin 8F10 T cell help is provided, with the caveat that Ab allotype was not investigated to confirm whether these insulin autoantibodies were coming from transgenic or endogenous anti-insulin B cells (17).

IgM-restricted anti-insulin B cells in V_H_125Tg.NOD mice are able to form spontaneous GCs (44). We found spontaneous anti-insulin GCs in the pancreatic lymph nodes of 8 of 14 class-switch competent V_H_125^SD^.NOD mice surveyed in this study (Fig. 1C). This contrasts with a prior study in which anti-insulin GCs were not noted in this model (17). In this study, we analyzed the frequency of GC+ cells among the insulin-binding gate, whereas the study by Wan et al. (17) reported the frequency of insulin-binding B cells among the GC gate. Our gating strategy more sensitively addresses the fate potential of insulin-binding B cells by eliminating the large (97–99%) competing repertoire of non-insulin-binding B cells that could be recruited into spontaneous and perhaps T1D-irrelevant GCs, whereas the alternative gating strategy is best suited to address the overall composition of GC B cells, which was massively perturbed when anti-insulin 8F10 T cell help was provided (17). Thus, our findings do not contradict this prior study, but rather represent an alternative analytic method that addresses a different question.

Pancreatic draining lymph nodes are sites important in the priming of autoreactive B and T cells during T1D development; their removal in young mice prevents diabetes (45). Mesenteric lymph nodes, however, are more important for intestinal immunity and are not sufficient to support diabetes in the absence of pancreatic lymph nodes. Increased activated caspase 3 expression was apparent in anti-insulin GC B cells compared with non-insulin-binding GC B cells that form spontaneously within the mesenteric lymph nodes; yet, no significant difference was observed in anti-insulin GC B cells in the T1D-relevant pancreatic draining lymph nodes. This could be due to differences in both the nature and magnitude of GC initiation in each site, variation in the quality of cognate T cell help provided, and/or tissue milieu–specific differences that diminish anti-insulin B cell apoptosis in pancreatic lymph nodes.

In contrast to blunted TD anti-insulin Ab responses when SRBCs and CFA were used as adjuvants, TI immunization with insulin BRT led to robust anti-insulin IgG production (Figs. 2–4). The frequency and number of insulin-specific GC B cells was greater after immunization with insulin BRT than with B:9-23 in CFA/IFA (CFA TD mean percentage ∼1% versus TI mean percentage ∼14%; CFA TD mean ∼120 cells versus TI mean ∼35,000 cells; Fig. 3 versus Fig. 4), whereas a comparable frequency of insulin-binding B cells adopted a GC phenotype following insulin SRBC immunization (SRBC TD mean percentage ∼12% versus TI mean percentage ∼14%), albeit with a reduced number of insulin-binding GC B cells relative to insulin BRT–immunized mice (SRBC TD mean ∼11,000 cells versus TI mean ∼35,000 cells; Fig. 2 versus Fig. 4). Our present study did not address whether the anti-insulin GC B cells (or limited insulin Ab produced) following insulin SRBC immunization were transgene derived, whereas TI immunization with insulin BRT is known to drive transgenic anti-insulin B cells to enter GCs and produce insulin Ab (28). Immunization and boost with B:9-23 in CFA/IFA failed to induce anti-insulin B cell expansion or GC formation, because both the frequency of anti-insulin B cells and the absolute number of GC B cells were comparable to those found in nonimmunized, spontaneous, V_H_125^SD^.NOD mice (Fig. 3 versus Fig. 1).

BRT is a type 1 TI Ag and has been shown to drive class-switch recombination of anti-insulin B cells in nontransgenic NOD mice (27) and in C57BL/6 mice that contain the V_H_125^SD^ BCR transgene (28). Insulin and BRT must be conjugated to drive anti-insulin GC B cell formation and anti-insulin IgG production, which is not elicited by mixing insulin and BRT (28). This suggests that costimulation of both the BCR and TLRs is required to break tolerance during TI immunization. BCR/TLR costimulation has been shown to induce TI class-switch recombination through the noncanonical NF-κB pathway (46). BCR signaling synergizes with TLR signaling for induction of activation-induced cytidine deaminase and Ig class switching through the noncanonical NF-κB pathway (47). Additionally, insulin BRT immunization also breaches immune tolerance in nonautoimmune prone V_H_125^SD^.B6 mice, which developed robust phenotypic and anatomical GCs and anti-insulin IgM and IgG2a production (28). Marginal zone B cells are thought to be strong contributors to TI responses (48). Anti-insulin B cells in V_H_125^SD^.NOD mice skew away from follicular B cells and toward marginal zone B cells (15), perhaps helping to explain their robust TI responses to insulin BRT.

Interestingly, although the expression of activated caspase 3 was elevated in anti-insulin B cells compared with foreign Ag-binding or non-antigen-binding B cells after CFA immunization, the average MFI of activated caspase was similar between anti-insulin B cells and non-insulin-binding B cells after insulin SRBC and insulin BRT immunization. It is possible that the potency of both SRBC and BRT adjuvants to stimulate anti-insulin GC B cells relative to CFA may reflect differences in whole insulin protein stimulation of anti-insulin BCRs with insulin BRT and insulin SRBCs, as opposed to insulin peptide CFA, which should not invoke BCR stimulation and downstream signaling. In support of this, insulin CFA immunization of nonautoimmune V_H_125^SD^/C57BL/6 mice elicits robust anti-insulin GC B cell formation in the absence of detectable anti-insulin Ab (unpublished data). Future studies are required to address whether anti-insulin B cells from V_H_125^SD^/NOD mice behave in a similar manner.

SRBCs are a classic TD adjuvant, and their potency to induce GC responses was recently linked to SRBC RNA activation of the RIG-1-like receptor MAVS (mitochondrial antiviral signaling) adapter pathway, post-phagocytosis, and downstream TLR3/7 stimulation. This cascade promotes the maturation of APCs via upregulation of surface costimulatory receptors and IFN-γ expression that help support GC formation (49). The potency of the SRBCs relative to CFA may also be from a combination of the MAVS signaling cascade and whole human insulin rather than peptides in CFA, which enables engagement of the BCR rather than direct peptide loading of MHC class II. A fraction of BCRs on anti-insulin B cells are loaded with endogenous (mouse) insulin that is present physiologically in the circulation (22, 24). Thus, another possible explanation for the small increase in anti-insulin IgG produced by immunization with SRBCs alone may be the fact that SRBCs alone provided enough stimulation to promote the expansion of already existing anti-insulin GCs/anti-insulin B cells that were receiving BCR stimulation from endogenous (circulating) insulin.

The inability of anti-insulin B cells to produce insulin autoantibodies following TD immunization may be the result of a failure of cognate CD4^+^ T cell help. Anti-insulin B cells in “double transgenic” V_H_125^SD^-8F10.NOD mice showed a dramatic increase in the proportion of GC B cells that recognized insulin, highlighting anti-insulin B cells as “fence sitters” that could be strongly recruited into GCs by pathologic anti-insulin 8F10 T cell clones that can give them the proper “push” (17). The 8F10 T cells are diabetogenic T cells that express an anti-insulin TCR and a critical T cell clone that drives T1D development in NOD mice (20, 50). However, crossing IgM-restricted V_H_125Tg.NOD mice with 2H6 anti-insulin TCR transgenic NOD mice results in a decreased proportion of anti-insulin B cells, as well as decreased anti-insulin B cell expression of costimulatory molecules and differentiation into GC B cells (44). Thus, the ability of anti-insulin B cells to enter GCs and form Ab may be dependent on both the number of cognate CD4^+^ T cells and the quality of help received.

TD immunization against insulin CFA elicits insulin-binding GC B cells but drives their increased expression of the apoptosis marker, activated caspase 3, in comparison with NP-binding B cells driven by simultaneous TD immunization against the foreign Ag, NP-KLH-CFA. This joint immunization strategy ensures that B cells responding to self (insulin) and foreign (NP-KLH) Ag are exposed to the same inflammatory milieu in a given mouse. Apoptosis is an important component of tolerance and a healthy GC response, occurring in both the light zone and dark zone of the GC (34, 51). Dysregulation of GC apoptosis through deletion of EAF2 (ELL-associated factor 2) promotes production of spontaneous autoantibodies and exacerbated collagen-induced autoimmune arthritis (52). Inability to regulate apoptosis within B cells via B cell–specific deletion of the apoptosis-associated protein Bim results in the development of spontaneous autoimmune phenotypes that resemble systemic lupus erythematosus, Sjögren’s syndrome, and rheumatoid arthritis (36). Our finding that activated caspase 3 expression is increased within insulin-binding (relative to NP-binding) GC B cells following CFA immunization suggests that insulin-binding B cells in V_H_125^SD^.NOD mice have an intact regulatory checkpoint that, under the right circumstances, can enhance anti-insulin GC B cell apoptosis. Future studies will be required to determine if this is impacted by the quality of BCR signaling that occurs during insulin immunization.

Additional reports provide evidence that regulation of autoreactive B cell maturation in GCs can limit the development of memory B cells and plasma cells. Mice that constitutively express the prosurvival protein, Bcl2, only in B cells that have expressed activation-induced cytidine deaminase have decreased expression of activated caspase 3, higher numbers of mature plasma and memory B cells, produce autoantibodies, and spontaneously develop glomerulonephritis with associated moribundity, suggesting that Bcl2-mediated release of GC apoptosis enhances spontaneous autoimmunity (35). 2-12H preplasma cells that recognize the anti-ribonucleoprotein Smith autoantigen undergo increased apoptosis compared with non-Smith-binding preplasma cells in a nonautoimmune mouse strain (53), consistent with the phenotype we observed in anti-insulin GC B cells in this study. However, when the 2-12H transgenic BCR was expressed on the lupus-prone MRL/lpr strain, apoptosis normalized and anti-Smith Ab secretion was restored and was linked with increased expression of the prosurvival receptor, BCMA, which binds BAFF and APRIL, relative to nonautoimmune mice (53). A preplasma cell block could be occurring within our V_H_125^SD^.NOD mice to limit insulin Ab production. However, anti-Smith B cells did not show any signs of participating in GC interactions; thus, preplasma cells in that model may arise through extrafollicular responses. Although systemic lupus erythematosus is an autoantibody-dependent disease, T1D is not (54, 55).
